# Study on Phenol–Formaldehyde Resin–Montmorillonite Impregnation and Compression Modification of Chinese Fir

**DOI:** 10.3390/polym16101385

**Published:** 2024-05-12

**Authors:** Lei He, Qian Zhang, Bengang Zhang, Hongyun Qiu, Jie Guo, Fengwen Sun, Zengfu Jiang, Weidong Zhou

**Affiliations:** 1 Forest Products Industry Research Institute, Jiangxi Academy of Forestry Sciences (Nanchang), Nanchang 330013, China; helei19842003@163.com (L.H.); bengang.zhang@univ-lorraine.fr (B.Z.); qiuqiuiuu1307@163.com (H.Q.); 17770850699@163.com (J.G.); 2 College of Materials Science and Engineering (Nanjing), Nanjing Forestry University, Nanjing 210037, China; zhangqianlulu@126.com; 3 Actel New Materials (Nanjing) Co., Ltd., Nanjing 210047, China; zengfu.jiang@aica-ap.com (Z.J.); weidong.zhou@aica-ap.com (W.Z.)

**Keywords:** Chinese fir wood, phenol–formaldehyde resin, montmorillonite solution, impregnation compression, compression rebound rate

## Abstract

In this study, a phenol–formaldehyde resin–montmorillonite intercalation composite solution was used as a modifier to treat Chinese fir via impregnation and compression. The basic characteristics and wettability of the PF (phenol–formaldehyde)–montmorillonite impregnation solution were analyzed. The effects of the solid content of PF, the quantity of montmorillonite, and the impregnation time on the impregnation weight gain of Chinese fir were studied through orthogonal experiments. The results showed that when the amount of montmorillonite was 1%, the wettability of the PF–montmorillonite impregnation solution performed the best, the curing time was short, and the curing strength was high. The optimal impregnation process consists of a PF solid content of 25%, an impregnation time of 120 min, and a montmorillonite ratio of 1%. Under these conditions, the modified Chinese fir was prepared via hot pressing. The effects of the addition of montmorillonite and different levels of compressibility on the physical and mechanical properties of modified wood were studied. The physical and mechanical properties were found to be better when the compression ratio was 33%: the density increased from 0.33 g/cm^3^ to 0.58 g/cm^3^; the surface hardness increased from 33.6 HD to 70.9 HD; the static bending strength increased from 60.4 MPa to 98.7 MPa; and the elastic modulus increased from 6 390 MPa to 11 498 MPa. After 30 days of release, the compression rebound rate was 3.97%. Meanwhile, the micromorphology and heat resistance of the impregnated compressed Chinese fir showed that the PF–montmorillonite impregnation solution entered into the cell cavity and intercellular space of the Chinese fir and formed a good composite, thus improving the water resistance, heat resistance, and physical and mechanical properties.

## 1. Introduction

Wood is a green renewable and reusable material, often used to make wood building materials (such as sawn timber, wood-based panels, composite lumber, flooring, etc.), pulp and paper, environmentally friendly fuel pellets (for heating), and charcoal [1,2]. Many wood species have long growth cycles, so fast-growing wood is favored by factories. In order to improve the properties of fast-growing wood and make up for its natural deficiencies, wood modification has become a major research focus [3,4,5].

Chinese fir (CF) is an exclusive, artificially fast-growing forest species in China, with widespread distribution across the provinces and regions in the south of the country. It possesses ecological characteristics such as water conservation and carbon sequestration [6], along with rapid growth and high yield. The total area of artificial Chinese fir forests in China is now 9.9 million hectares, with a storage volume of 755 million cubic meters. This constitutes one-fourth and one-third of the overall area and storage volume of artificial tree forests in China, respectively, securing the first position according to the Ninth National Forest Resources Inventory [7]. The low density, strength, and hardness of this species, along with the poor dimensional stability of fast-growing artificial Chinese fir forests, have restricted its widespread industrial usage [8]. Therefore, the research focus on how Chinese fir processing has shifted towards modification to broaden its range of applications.

Currently, the modification methods for Chinese fir are primarily categorized as reactive and non-reactive, based on whether the modifier’s active groups chemically react with the main wood components [9]. Reactive modification enhances the size, stability, and physical and mechanical properties of Chinese fir, but the sustainable performance of the modifier is crucial. On the other hand, non-reactive modification induces physical changes such as compression or swelling, yet its stability and effectiveness are inferior to reactive modification. Hence, integrating reactive and non-reactive methods for Chinese fir modification enhances its dimensional stability and physical/mechanical properties while minimizing modifier loss. Nano-montmorillonite (MM) is a silicate layered structure comprising two layers of silicon oxide tetrahedral wafers sandwiched between an aluminum oxide octahedral wafer, with the layers connected by oxygen atoms [10]. Nano-montmorillonite exhibits excellent water–heat combination characteristics due to the high stiffness of its silicate layers [11]. The modification process involves blending phenolic resin with a nano-montmorillonite aqueous solution and intercalating it into the silica interlayer of the nano-montmorillonite to obtain a modified solution that integrates polymer materials and inorganic crystals. This solution can penetrate the intercellular layer and cell cavity of Chinese fir [12]. After impregnation, the Chinese fir undergoes high-temperature and high-pressure densification treatment [13]. First, the phenolic resin–montmorillonite-modified liquid effectively penetrates the Chinese fir cells, undergoing cross-linking reactions with wood components. Second, after entering the wood, the modified liquid solidifies and forms phenolic resin under high temperature and pressure, enhancing the anti-loss property. The composite effect significantly enhances the performance of Chinese fir [14].

The authors of this study utilized artificial Chinese fir as the raw material, prepared a phenolic resin–montmorillonite-modified solution, and subjected the Chinese fir to immersion under room temperature and normal pressure. Subsequently, the immersed Chinese fir underwent curing through hot pressing with phenolic resin. By analyzing the impact of montmorillonite addition on the properties of the immersion-modified solution, the study identified and selected the optimal immersion process. The study examined the dimensional stability and the physical and mechanical properties under various compression ratios and analyzed the corresponding change in the rebound rate. The study conducted tests on the performance of the modified Chinese fir, analyzed the underlying mechanism, and aimed to provide references for the modification and high-value utilization of Chinese fir.

## 2. Materials and Methods

### 2.1. Materials

The authors used 20-year-old Chinese fir (*Cunninghamia lanceolata*) sapwood, with a moisture content of 8~10%.

A high-purity sodium-based montmorillonite (model BP-652, purchased externally) with interlayer cations of sodium ions and a spacing of 1.3 nm was used. It was off-white or beige in color, non-toxic, and odorless, and was obtained as a soft powder with good dispersibility in water. 

The phenol–formaldehyde resin (PF) used in this study was produced by Aiketeer New Materials (Nanjing) Co., Ltd. (model 4980, Nanjing, China), and was obtained as a red-brown uniform transparent solution with a solid content of 58%, a viscosity of 612 mPa·s, and a relative molecular weight of 400 g/mol.

### 2.2. Modified Solution Preparation

Montmorillonite solutions of 1%, 2%, and 3% were prepared via dilution in water. Each solution was stirred at 900 r/min for 30 min at room temperature to achieve a uniformly dispersed phase. Simultaneously, the PF was diluted to achieve a solution with a solid content of 25%. The montmorillonite aqueous solution was thoroughly mixed with the diluted PF solution to create a modified solution.

### 2.3. Modified Fir Preparation

The fir was cut into 20 cm × 5 cm × 1.5 cm (L × R × T) specimens, without end-sealing treatment. After drying in an oven at 103 °C until completely dry, the specimens were placed in a dryer for 15 min; the mass was weighed and recorded as *m*1. Then, the fir specimens were placed in a modified solution and immersed for 60, 90, and 120 min. The test piece was removed and drained until no liquid drips were visible on the surface. The surface was then wiped and the mass weighed again, this time being recorded as *m*2. The equation for calculating the dry weight gain rate (*WGR*) is as follows, Equation (1):

(1) $$WGR=\frac{\left(m2-m1\right)}{m1}\times 100\%\times C$$

where *C* is the modified liquid solid content, %.

Under normal temperature and pressure, the PF solid content, fir infusion time, and montmorillonite addition were taken as the influencing factors, and the dry weight gain rate after fir infusion was taken as the assessment index. Orthogonal experiments were designed according to the parameters outlined in Table 1 to optimize the infusion process. The number of replicates for each group of specimens was 3, and the dry weight gain rate was taken as the average value of 3 specimens.

The specimens subjected to the optimized immersion process underwent drying in an oven at 60 °C until achieving a moisture content of 8%. Subsequently, the samples were placed in a hot press at 130 °C, with a hot-pressing time of 1.2 min/mm, a unit pressure of 15 MPa, and compression to thicknesses of 1.2 cm (compression rate of 20%), 1.0 cm (compression rate of 33%), and 0.7 cm (compression rate of 53%) using a thickness gauge.

### 2.4. Performance Testing

#### 2.4.1. Performance of Modified Liquid

The viscosity, pH value, water mixability, and other indicators of the modified liquid were tested according to GB/T 14074-2006 “Test Methods for Wood Adhesives and their Resins” [15].

A contact angle tester was used to analyze the change in the static contact angle of the dipping solution in the cross and tangential face of the fir wood. The test conditions included thermostating the samples at 25 °C for 4 h with a droplet volume of 3 μL.

#### 2.4.2. Properties of Modified Fir in Optimal Treatment Conditions 

The density, static flexural strength, modulus of elasticity, and water-absorbing thickness expansion rate of the specimens were tested with reference to GB/T 1933-2009 Method for Determination of Density of Wood [16], GB/T 1936.1-2009 Method for Determination of Flexural Strength of Wood [17], GB/T 1936.2-2009 Method for Determination of Flexural Modulus of Wood [18], and GB/T1934.2-2009 Method for Determination of Wet Expansion of Wood, respectively [19]. The hardness was tested using a Shore hardness tester. The compression resilience was tested by keeping the specimens at room temperature and measuring the thickness changes after 1, 7, 14, and 30 days, respectively, and calculating the resilience S according to the following, Equation (2) [20]:

(2) $$S=\frac{d_{2}-d_{1}}{d_{0}-d_{1}}\times 100\%$$

where d_0_ is the thickness of the specimen before compression, mm; d_1_ is the thickness of the specimen after compression, mm; and d_2_ is the thickness of the specimen after rebound, mm.

Wetting performance: Deionized water was used as the liquid phase to test the surface wettability of the composite using an optical contact angle measuring instrument (Model: HARKE-SPCAX3). The contact angle of the blend was determined by drawing the contact angle change curve and observing the number of contact angles displayed after it becomes stable. Each sample was tested 5 times, and the average value was used as the final result.

Micromorphological analysis: The distribution of the modified liquid in the fir cells was observed using a scanning electron microscope (Model: Czech TESCAN MIRA LMS), and the cross, radial, and tangential sections of the specimens were observed, respectively. The specimens were then fixed on the carrier stage with double-sided adhesive and sprayed with gold (Model: Quorum SC7620), and the test voltage was 15 kV. 

Thermogravimetric analysis: The thermogravimetric analysis of the fir material and compression-modified fir formulated using the optimized dipping process was carried out via TGA (Model: NETZSCH STA 449 F3, Germany), using two samples each before and after modification, with sample sizes ranging from 10 to 20 mg, and under the following conditions: a nitrogen flow rate of 40 mL/min; a heating rate of 10 °C/min; and a measurement range of 30 to 1000 °C (room temperature to 1000 °C).

## 3. Results and Discussion

### 3.1. Effect of Montmorillonite Addition on the Performance of Modified Liquids

#### 3.1.1. Basic Properties of Modified Liquid 

The viscosity of the modified liquid increased with an increase in the addition of montmorillonite (Table 2). The pH value and free aldehyde did not change significantly. The water solubility times could not be measured, mainly because the montmorillonite and PF resin mixed together to form opaque liquids, thus affecting the judgment of the turbidity point. 

#### 3.1.2. Wetting Performance

**Figure 1:**
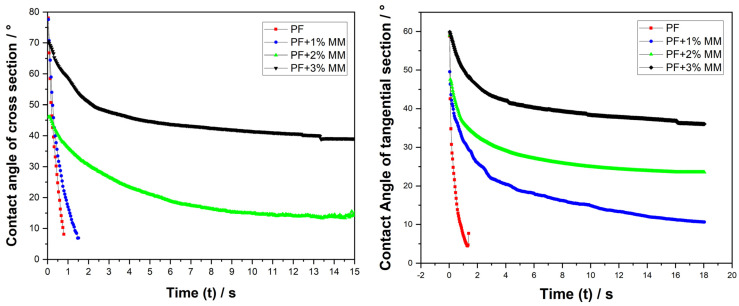
Dynamic contact angle of impregnation solutions with Chinese fir.

The results in Figure 1 indicate that the PF dipping glue exhibits effective wetting on the fir. The contact angle in both the cross section and tangential section decreased to below 10° within 1–2 s. The wetting performance of the PF dipping glue with 1% montmorillonite remained superior in the cross section, requiring approximately 2 s for the contact angle to decrease below 10°. However, the contact angle in the tangential section was less favorable than in the cross section due to an evidently less porous structure. With the addition of 2% montmorillonite to the PF dipping glue, the wetting property notably decreased. It took approximately 18 s for the contact angle to fall below 10° in the cross section. Beyond 18 s, the contact angle continued to decrease to below 10°. Following the addition of 2% montmorillonite to the PF infusion, the wettability markedly decreased. It required more than 15 s for the contact angle in the cross section to decrease to 10°, while that in the tangential section did not fall below 10°. Upon the addition of 3% montmorillonite to the PF infusion, the contact angle in both the cross and tangential sections remained above 40° after 15 s. This suggests that the wettability of the PF infusion on the fir remained relatively good, taking more than 2 s to reduce the contact angle in the tangential section to below 10°, indicating the poor wettability of the fir.

### 3.2. Optimization of the Impregnation- and Compression-Modified Fir Process

#### 3.2.1. Optimization of the Dipping Process

The results in Table 3 and Table 4 illustrate that, among the three factors investigated in the experiment, PF solid content (A) had the most significant impact on the dry weight gain rate, followed by montmorillonite content (C) and dipping time (B). Within the experimental range, the dry weight gain rate initially increased and then decreased with the rise in the PF solid content. Montmorillonite content had the second-largest effect on the dry weight gain rate, while dipping time showed no significant impact. The influence of dipping time on weight gain was not significant. The optimal combination was determined to be A2B3C1, signifying that the maximum dry weight gain rate was achieved with a PF solid content of 25%, a dipping time of 120 min, and a montmorillonite addition of 1%. This aligns with the findings of the contact angle test. Repeatability tests conducted under the preferred process conditions resulted in an average dry weight gain rate of 12.54% (the average value obtained after repeated testing under the conditions of the optimal combination A2B3C1), indicating excellent experimental repeatability. Additionally, based on the results of the dry weight gain rate test in the control group (PF resin modifier without the addition of montmorillonite), the dry weight gain rate in each group of montmorillonite-added infusions exceeded that of the pure PF infusion under the test’s selected conditions. This suggests that the combination of montmorillonite and PF contributed to an increased dry weight gain rate.

#### 3.2.2. Impact of Compression Rate on the Physical and Mechanical Properties of Modified Fir Wood

Under the optimal conditions of the infusion process, the dry weight gain rate reached 12.36%. The fir wood underwent infusion and hot compression treatment, blending the PF–montmorillonite with the pure PF modification solution, resulting in the creation of infusion-compression-modified fir wood. Table 5 reveals that the water-absorbing thickness expansion rate of the modified fir increased with the rise in the compression rate. All of the physical and mechanical properties of the modified fir showed improvement, with the most favorable outcomes observed at a compression rate of 33%. Specifically, the density increased from 0.328 g/cm^3^ to 0.581 g/cm^3^, the surface hardness rose from 33.6 HD to 70.9 HD, the static flexural strength increased from 60.4 MPa to 98.7 Mpa, and the modulus of elasticity rose from 6390.8 Mpa to 11,498 Mpa. At a compression rate of 53%, the physical and mechanical properties were inferior to those observed at a 33% compression rate. Although there was improvement compared to the unmodified fir, bulging occurred due to the excessively high compression rate.

Simultaneously, the incorporation of montmorillonite enhanced the physical and mechanical properties of the modified wood. The modified fir with added montmorillonite exhibited higher static flexural strength and modulus of elasticity compared to the pure PF-impregnated and compressed fir. At a low compression rate (20%), the density and surface hardness of the PF–montmorillonite-modified fir were lower than those of the pure PF-modified fir. This is attributed to the PF resin’s enhanced fluidity under low compression rates, allowing better filling of the fir material. The resulting shrinkage stress was more effectively released. However, the PF–montmorillonite-modified liquid faced challenges in compressing into cellular pore spaces due to poor penetrability, resulting in lower density and hardness values. As the compression rate increased, the water absorption thickness expansion rate of the pure PF-modified fir surged, leading to severe bulging at a compression rate of 53%. This is because pure PF-impregnated modified fir experiences significant shrinkage and dehydration during the hot-pressing process, generating substantial shrinkage stress and promoting bulging and poor adhesion. Montmorillonite addition fills the voids resulting from condensation and dehydration, providing an effective buffering effect. Additionally, the chemical bond formation between the PF–montmorillonite and fir closes some hydrophilic groups, enhancing water resistance. At a compression rate of 33%, the PF–montmorillonite-modified fir surpassed the pure PF-modified fir in terms of physicomechanical properties and dimensional stability.

As can be seen from Table 6, the fir material underwent impregnation with the phenolic resin–montmorillonite-modified liquid at high temperature and pressure. While a small portion of the modified liquid was extruded, most of it cured and molded within the fir cells. The loss of modified liquid was 1.35%, resulting in the stable performance of the modified fir. Simultaneously, the compression rebound rate of the modified fir increased with time and reached equilibrium in approximately 14 days. The lower the compression rate, the lower the compression resilience at the final equilibrium. As the compression ratio decreased, the compression resilience at the final equilibrium also decreased. After 30 days of aging, the compression resilience of the modified fir wood with a 33% compression ratio was 3.97%.

### 3.3. Analysis of Immersion–Compression Modification Mechanism

#### 3.3.1. Micromorphological Analysis

**Figure 2:**
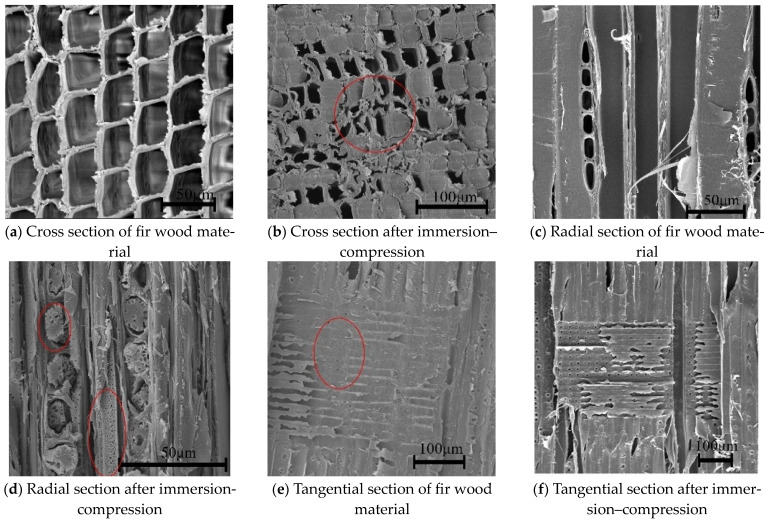
Microstructure of Chinese fir before and after impregnation compression.

The microstructures of three sections of fir (Figure 2a,c,e) reveal typical features of coniferous wood. Axial tubular cells are predominantly distributed in the cross section, while thin-walled ray tissues are prevalent in the tangential and radial sections. An examination of Figure 2b,d,f reveals the presence of liquid on both axial tubular cells and ray thin-walled tissues after PF–montmorillonite leaching. This suggests that the leaching liquid penetrated the cell lumen and interstitial space of the fir. The inner walls of the tubular cells and ray cells were coated with cured phenolic resins and nano-montmorillonite, forming a dense filling. This indicates the successful infiltration of the PF–montmorillonite modification liquid into the interior of the wood, effectively enhancing the density and the physical and mechanical properties of the modified fir.

#### 3.3.2. Thermal Performance Analysis

**Figure 3:**
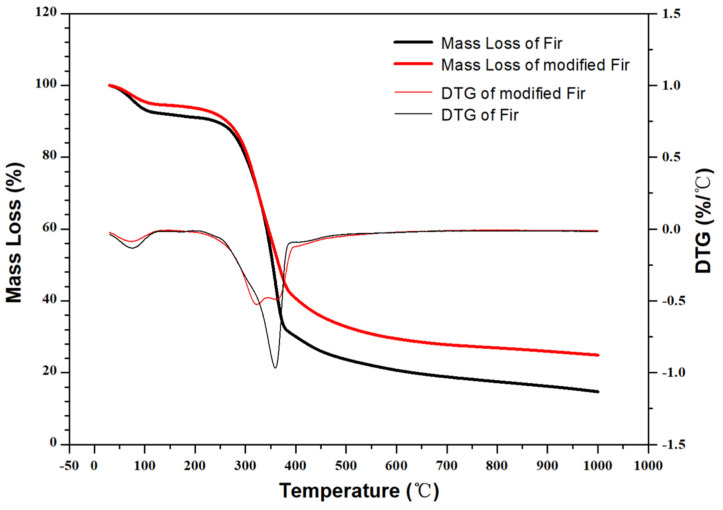
Thermogravimetric analysis of Chinese fir before and after impregnation and compression.

Figure 3 shows the changes in the thermal performance of the fir before and after the dipping–compression modification, divided into three stages. The first stage (30~130 °C) involves water evaporation produced by the weight loss; the loss rate is less than 10%. In this stage of modification, the weight loss is less, because in the dipping–compression of the fir wood after the cellular cavities are densely populated, the water absorption energy decreases and the amount of water is reduced. The second stage (130~400 °C) mainly involves changes in the wood chemical composition, as well as hemicellulose and cellulose decomposition [21,22]. The wood experiences a heat loss of 62% in addition to the decomposition of wood components, but there is also thermal decomposition of the PF at 345 °C [23]. Moreover, due to the overall heat loss from 62% to 53%, the resin’s skeleton structure is destroyed, and the molecular chain is fractured. The third stage (400~1000 °C) is the combustion stage [24]: the pyrolyzed products further decompose to produce CO_2_, alkanes, and other gases, and at this time, the heat loss before and after the modification of the change is not significant at approximately 15%. The final residual carbon rate is 14.9% for the wood material and 24.88% after modification, indicating that the inorganic montmorillonite that filled the interior of the wood reduces the combustion rate, thereby helping to promote char formation. The above results indicate that the PF–montmorillonite infusion–compression modification improved the heat resistance of the fir wood.

## 4. Discussion

Previous methods of modifying Chinese fir are mainly divided into reactive modification and non-reactive modification. Reactive modification enhances the size, stability, and physical and mechanical properties of Chinese fir, but the sustainable performance of the modifier is crucial. On the other hand, non-reactive modification induces physical changes such as compression or swelling, yet its stability and effectiveness are inferior to reactive modification. Hence, integrating reactive and non-reactive methods for Chinese fir modification enhances its dimensional stability and physicomechanical properties while minimizing modifier loss. In this study, Chinese fir was used as the raw material. A phenolic resin–montmorillonite-modified solution was prepared, and the Chinese fir was subjected to immersion under room temperature and normal pressure. The addition of montmorillonite reduced the wettability of the modification solution. It helped to improve the water resistance of the modified solution and extend its service life. After modification, the density, strength, hardness, and dimensional stability of the fir wood were all improved compared with those before modification. This broadens the application scope of Chinese fir and improves the application value of Chinese fir. However, PF resin is not very friendly to the environment. Therefore, developing environmentally friendly resin-modified fir while maintaining good performance is a future research direction.

## 5. Conclusions

1. Our results show that the addition of montmorillonite had a small effect on the heat-curing performance of the modified liquid, but it increased the viscosity of the modified liquid and reduced the wettability. When the amount of montmorillonite added was 1%, the modified liquid had the best wettability on the fir wood, and the infiltration could be completed in 18 s. The curing time was 17.5 min, and the maximum energy storage modulus after curing was 1.8 × 10^7^ Pa.
2. The influence on the dry weight gain rate was in the order of montmorillonite content > PF solid content > dipping time. The optimal process was 25% of the PF solid content and 120 min of dipping time, and the dry weight gain rate reached 12.54% when the montmorillonite additive amount was 1%.
3. Under the optimal PF–montmorillonite impregnation process, the impregnated Chinese fir was hot-pressed. The physical and mechanical properties were the best when the compression rate was 33%. Compared with ordinary Chinese fir, the density increased from 0.35 g/cm^3^ to 0.58 g/cm^3^, and the surface hardness increased from 37.9 HD to 70.9 HD. The static bending strength increased from 73.4 MPa to 98.7 MPa, the elastic modulus increased from 8640.7 MPa to 11,498 MPa, and the compressive rebound rate was 3.97% after aging for 30 days. Compared with pure PF impregnation–compression-modified Chinese fir, all of the detection properties were improved.
4. The PF–montmorillonite solution enters the cell cavity and cell interstices of the fir, and the PF, montmorillonite, and fir form a certain chemical combination that plays the role of filling and densification, thus improving the water resistance, heat resistance, and physical and mechanical properties of the fir. Compared with ordinary fir, the overall heat loss of the impregnation–compression-modified fir was less than 10 percent, and the final residual carbon rate increased from 14.9 percent to 24.88 percent.

## Figures and Tables

**Table 1:** Orthogonal factors.

Level	Influence Factor			Assessment Indicators
	PF Solid Content/%	Infiltration Time/min	Montmorillonite Addition/%	
1	20	60	1	Dry weight gain rate
2	25	90	2	
3	30	120	3	

**Table 2:** Basic characteristics of the immersion solution.

Montmorillonite Addition/%	Viscosity/mPa s	pH Value	Water Hybrid	Free Aldehyde/%	Initial Exothermic Temperature/°C	Maximum Exothermic Temperature/°C
0	15.5	9.81	>20	0.056	118.07	157.05
1	506	9.68	-	0.055	119.59	155.81
2	1118	9.70	-	0.055	119.76	155.83
3	1916	9.58	-	0.054	119.16	155.82

**Table 3:** Result of orthogonal trial.

Test Number	Solid Content (%) A	Infiltration Time (min) B	Montmorillonite Addition (%) C	Mean Value of Dry Weight Gain Rate by Immersion (%)
1	1	1	1	8.58
2	1	2	2	7.30
3	1	3	3	8.18
4	2	1	2	9.45
5	2	2	3	9.35
6	2	3	1	12.11
7	3	1	3	8.98
8	3	2	1	11.35
9	3	3	2	9.08
Control group	2	3	0	6.54

**Table 4:** Analysis of variance.

		Variance Analysis			
Source of Variance	Sum of Squared Deviations	Degrees of Freedom	Sum of Mean Square	F	*p*-Value
A	25.93	2	12.96	12.87	0.09
B	2.81	2	1.40	1.39	0.01
C	23.24	2	11.62	11.54	0.08

F_0.1_ (2, 2) = 9; F_0.05_ (2, 2) = 19; F_0.01_ (2, 2) = 99.

**Table 5:** Physical and mechanical properties of impregnated and compression-modified Chinese fir.

Compression Ratio (%)	Modified Leaching Solution	Absorbent Thickness Expansion/%	Density/(kg·m^−3^)	Surface Hardness/HD	Static Bending Strength /MPa	Modulus of Elasticity /MPa	Note
20%	PF–montmorillonite Modified liquid	8.3	0.500	69.8	82.4	10 866.0	
	PF	3.1	0.523	74.1	78.7	10 822.3	
33%	PF–montmorillonite Modified liquid	11.6	0.581	70.9	98.7	11 498.0	
	PF	26.8	0.592	83.3	90.6	9 876.6	
53%	PF–montmorillonite Modified liquid	12.3	0.538	53.8	86.5	9 424.0	burst
	PF	-	-	-	-	-	severe bulging
Uncompressed	-	-	0.328	33.6	60.4	6390.8	

**Table 6:** Change in compression resilience of impregnated and compressed Chinese fir.

Compression Ratio/%	Compression Resilience/%			
	1 Day	7 Days	14 Days	30 Days
20	0.20	1.29	3.03	2.89
33	0.10	1.43	3.81	3.97
53	0.15	1.80	5.27	5.45

## Data Availability

The original contributions presented in the study are included in the article, and further inquiries can be directed to the corresponding author/s. The raw data supporting the conclusions of this article will be made available by the authors on request.
